# The Etiology of Dysentery

**Published:** 1886-07

**Authors:** 


### The Etiology of Dysentery.

Drs. Cordorelli, Margheri and Aradas have recently published, in the *Rivista Int. di Med. and Chirurg*, the results of their investigations of the causes of epidemic dysentery.

In the fæces of patients affected with dysentery, were found various micro-organisms, which were discovered to belong to four classes—two known, the *bacterium termo and bacterium lineolum*, and two unknown. Their studies were confined to the two latter. Both formed round colonies of a yellowish color, and developed rapidly under a temperature of from 30° to 35° cent. (86 to 95 Fahr.) One of these bacteria is smaller than the b. termo, has no spores, and, besides the Brownonian movement, has a motion of its own. It may be stained by an aqueous solution of Fuchsin, or by methylene violet. The other may be colored in the same way, only the red of the Fuchsin becomes violet in about twenty-four hours. The form of the latter is rod-like, the larger ones having a spore at the extremity.

A small portion of microbes of the first variety was injected into rabbits. This was uniformly followed by a rise of temperature lasting twenty-four hours, but no lesion was discovered in any case. They accordingly called this micro-organism the *bacterium pyrogenum.*

In six rabbits, a cultivation of the other bacteria were injected. All died in from one to thirteen days. The intestines were found completely filled with a thick mucus, the bladder full of urine, the spleen black, and the intestinal vessels filled with dark blood. The six rabbits presented a clinical picture of acute epidemic dysentery.

The bacilli were not found in the blood, nor in the intestinal walls, but only in the secretion around ulcerated points. With a specially prepared apparatus, they sought for this microbe, which they have named the *bacillus dysentericus*, in the air of rooms in which dysenteric patients were lying, but failed to find it. But they did succeed in discovering it in water. This was cultivated, injected into rabbits, and produced the same symptoms as were developed in the six rabbits mentioned above.

These important discoveries will undoubtedly be applied in therapeutics. A reliable germicide must be obtained, which, if thoroughly applied, will work an infallible cure.

